# Arbovirus-Mosquito Vector-Host Interactions and the Impact on Transmission and Disease Pathogenesis of Arboviruses

**DOI:** 10.3389/fmicb.2019.00022

**Published:** 2019-01-23

**Authors:** Yan-Jang S. Huang, Stephen Higgs, Dana L. Vanlandingham

**Affiliations:** ^1^Department of Diagnostic Medicine/Pathobiology, College of Veterinary Medicine, Kansas State University, Manhattan, KS, United States; ^2^Biosecurity Research Institute, Kansas State University, Manhattan, KS, United States

**Keywords:** arthropod vectors, arboviruses, vertebrate hosts, infection, transmission

## Abstract

Hundreds of viruses, designated as arboviruses, are transmitted by arthropod vectors in complex transmission cycles between the virus, vertebrate host, and the vector. With millions of human and animal infections per year, it is critical to improve our understanding of the interactions between the biological and environmental factors that play a critical role in pathogenesis, disease outcomes, and transmission of arboviruses. This review focuses on mosquito-borne arboviruses and discusses current knowledge of the factors and underlying mechanisms that influence infection and transmission of arboviruses and discusses critical factors and pathways that can potentially become targets for intervention and therapeutics.

## Arboviruses, Transmission, and Human Diseases

The first revelation that mosquitoes can vector human pathogens was the discovery of mosquito transmission of filarial worms by Sir Patrick Manson (Manson, 1878). In 1881, Carlos Finlay hypothesized that the etiologic agent responsible for yellow fever might be carried by mosquitoes, and this theory was proven to be true by Major Walter Reed who, in 1900 made the first observation that a human virus, yellow fever virus (YFV), can be transmitted by mosquitoes. The concept and term of “arthropod-borne” virus transmission was first introduced to the field of virology in 1942 (Hammon and Reeves, 1945), and as pioneering discoveries are published, the term arthropod-borne virus, or arbovirus, continues to evolve. In the publication, “Arthropod-borne and rodent-borne viral diseases”, by the World Health Organization, members of its scientific group defined arboviruses as *“viruses that share the characteristic of being naturally maintained through biological transmission between susceptible vertebrate hosts by hematophagous arthropods or transovarial transmission from infected female arthropods to her progeny*” (WHO, 1985). Whilst mosquitoes are responsible for the transmission of many medically important arboviruses, other arthropod taxa play an important role in vectoring human viruses as well. For example, ticks, especially hard ticks under the *Ixodidate* family, are vectors for several tick-borne flaviviruses such as tick-borne encephalitis virus (TBEV), Powassan virus (POWV), and Omsk hemorrhagic fever virus, and bunyaviruses such as Crimean-Congo hemorrhagic fever virus.

Although human-to-human transmission through the exchange of infectious body fluids has been reported for several arboviruses, the majority of arthropod-borne transmission associated with human diseases is achieved by infected arthropods feeding on people. Often, as for example in the case of West Nile virus (WNV), the virus is transmitted by mosquitoes to reservoir and amplifying hosts that develop a viremia that is sufficient to infect mosquitoes, and humans, as dead-end hosts, become infected with potentially severe and potentially fatal disease. Under certain circumstances, for example when vectors and naive hosts are abundant, an enzootic cycle limited to a specific region or period may become epizootic with large numbers of cases. Because of the requirement for the virus to replicate in both the arthropod and vertebrate host, it is immediately apparent that the process of biological transmission creates multiple opportunities for interactions among vertebrate hosts, vectors, and viruses. These interactions can occur on multiple levels that can ultimately impact transmission patterns and disease pathogenesis. At the ecological level, transmission patterns of arboviruses can be influenced by the specific species of vector or vertebrate hosts involved in the transmission cycle. This is particularly true as the geographic distribution of vector species and arboviruses is often expanded through the movements of humans and cargo. This dispersal can lead to a change in the transmission patterns of arboviruses as they are introduced into new regions. A particularly good example is the spread of *Aedes aegypti* from Africa to the New World several hundred years ago. *Ae. aegypti* subsequently became responsible for the urban transmission of YFV in the Americas and continues to be a medically important species following the emergence of dengue virus (DENV), chikungunya virus (CHIKV), and Zika virus in the New World (Tabachnick, 1991; Gould et al., 2003). *Culex quinquefasciatus* was also introduced to the New World, presumably near the same time as *Ae. aegypti*, and has become an important vector species for WNV. The ongoing invasion of *Ae. albopictus* into various locations since the late 20th century has further created significant ecological and health threats because of its competence for various arboviruses (Vanlandingham et al., 2016). Although the introduction of competent vector species and pathogenic arboviruses into new geographic regions, where immunologically naïve hosts are present, can profoundly change the epidemiology of arboviruses, the change in epidemiology and overall disease burden often has little impact on disease pathogenesis in humans. In this review, we focus on the discoveries of interactions between arboviruses, vectors, and vertebrate hosts at the cellular and molecular levels, which ultimately changed transmission patterns and disease pathogenesis. Such discoveries have potential applications as targets for antiviral therapies, vaccines, or preventive interventions for arboviral diseases.

Of obvious relevance to geographic distribution is the effect of the environment on both the biology of the vectors but also the relationships between the vectors and the viruses. Climate, and particularly temperature and rainfall, have a significant effect on the distribution and abundance of different mosquito species. At higher temperatures, the mosquito life cycle is shorter than at lower temperatures, and typically there is a species-specific lower temperature threshold at which the species cannot survive. This in essence determines the geographic distribution of the species. For instance, *Ae. aegypti*, one of major vectors for arboviruses, is more sensitive to low temperature in nature than *Ae. albopictus*, limiting its geographic distribution to tropical and subtropical areas (Chang et al., 2007). Temperature also influences the kinetics of replication and dissemination of viruses in the mosquito and becomes a determinant for vector competence. Under laboratory conditions, extrinsic incubation period of arboviruses at higher temperatures is often shorter than at lower temperatures (Liu et al., 2017). In nature, the fluctuation of temperature has also been found to alter vector competence of disease vectors (Carrington et al., 2013a,b). As a consequence of environmental influence on the vector, outbreaks of arboviruses, for example dengue viruses, can be correlated with season, specifically those that promote high mosquito population densities (Campbell et al., 2013). As with the vector, the environment can influence distribution, abundance, and even susceptibility of vertebrate hosts. Effects could include migration patterns and reproductive status, and availability of young naïve hosts. One of the most aggressive dispersal of CHIKV in human history was attributed to an abrupt increase of *Ae. aegypti* population followed by emergence of the East-Central-South-African genotype of CHIKV in the coastal region of Kenya (Chretien et al., 2007). As a consequence of the outbreaks, millions of individuals were infected in the archipelago along the Indian Ocean between 2005 and 2006. Numerous attempts have been made to produce predictive models that relate environment/climate to disease outbreaks, perhaps the most successful being for Rift Valley fever virus (Linthicum et al., 1999). Environmental influences on arboviral infections and vectors has been reviewed by Mellor (Mellor, 2004). Although it is logical to suggest that if temperatures increase in specific areas, then one could anticipate some redistribution of, for example, tropical mosquitoes into traditionally temperate zones, and as a consequence, perhaps a geographic change in the distribution of associated arboviruses, but to date there seems to be no compulsive proof that this has happened (Gould and Higgs, 2009).

Regardless of the arbovirus or the vector species, a significant number of arboviral infections in humans are asymptomatic or cause only a mild transient fever. Arboviruses can also cause other non-specific symptoms which are observed in most viral infections, such as rash and myalgia. Specific clinical manifestations can be generally classified into distinct categories of clinical outcomes: hemorrhagic fever, encephalitis or central nervous system involvement, or arthritis (Barrett and Higgs, 2007; Higgs, 2008; Higgs and Vanlandingham, 2016). The possibility of further development of severe disease is dependent on the arbovirus and the physiological and immunological condition of the patient. Several groups of arboviruses can cause viral hemorrhagic fever including mosquito-borne flaviviruses under the YFV and DENV serocomplexes, tick-borne flaviviruses such as Omsk hemorrhagic fever virus and Kyasanur Forest disease virus, mosquito-borne bunyaviruses in the genus *Phlebovirus* such as Rift Valley fever virus, and tick-borne bunyaviruses in the genus *Nairovirus* such as Crimean-Congo hemorrhagic fever virus. The hemorrhagic form of the disease is likely to have the highest public health significance based on the case numbers. This is largely due to the increased disease burden created by DENV, which is the etiological agent for dengue hemorrhagic fever (Gubler, 1998).

Another important re-emerging mosquito transmitted pathogen is YFV, which has caused significant mortality in Africa since 2015 due to a shortage of the global stockpile of the 17D vaccine (The Lancet, 2016). The movement of viremic YF-infected people from Africa to China has caused concern that a yellow fever outbreak could occur in the immunologically naïve populations in Asia where the urban vector, *Ae. aegypti*, is present in large numbers (Calisher and Woodall, 2016). The other important form of severe disease in humans is encephalitis and related central nervous system diseases caused by several arboviruses from different virus families (Higgs and Vanlandingham, 2016). For example, members of flaviviruses within the Japanese encephalitis virus and TBEV complexes are known to cause encephalitic diseases in humans (Higgs and Vanlandingham, 2016). Similarly, infection of New World alphaviruses such as Venezuelan equine encephalitis virus and Eastern equine encephalitis virus also cause encephalitis in humans. Orthobunyaviruses, in the California serogroup, such as La Crosse virus, are etiological agents for pediatric encephalitis, especially in North America (Vasconcelos and Calisher, 2016). Distinct from the viral hemorrhagic fever diseases caused by systematic infection, lethal encephalitis is normally the consequence of infection taking place in an incidental host, which normally does not develop viremia high enough to sustain the transmission cycle but which often succumbs to disease (Weaver and Barrett, 2004). The third form of human disease caused by arbovirus infection is arthritis, which is mainly associated with the Old World alphaviruses, for example CHIKV (Suhrbier et al., 2012; Higgs et al., 2018). Although the disease is rarely lethal, it often leads to a significant disease burden in the population and substantial economic loss in endemic regions (Higgs et al., 2018).

## Virus-Vector Interactions

Infection of an arthropod vector is typically required to sustain the transmission cycle of arboviruses. This process can have a direct impact on the pathological outcome of arboviruses in humans or other vertebrate hosts and virus-vector interactions can often determine the epidemic potential of arboviruses (Schneider and Higgs, 2008). Approaches used to better understand virus-vector interactions can be multidisciplinary examining vector biology, molecular genetics, and arbovirology. Infection with a virus, for example WNV, can influence transcription of mosquito genes and subsequent protein abundance, for example in salivary glands, and can potentially influence virus infection and disease development in the vertebrate host (Girard et al., 2010). Effects of saliva on the vertebrate and its influence on virus infection of the vertebrate is discussed in more detail below. Recently, it has also become evident that the presence of the vector microbiome (symbiotic bacteria) persistently infecting mosquitoes can also alter virus-vector interactions and affect the infection process of arboviruses (van den Hurk et al., 2012; Dennison et al., 2014). Knowledge of virus-vector interactions is often helpful in identifying critical factors that assist in the development of new life-saving technologies, including vaccine candidates and novel control strategies.

### Cellular Factors Controlling Arbovirus Infections in Arthropods

Identification of intrinsic factors, for example immune-related genes that control the infection process of arboviruses in arthropods, has long been an important topic for arbovirus research (Sim et al., 2014). As demonstrated with vectors collected from different geographic regions, genetic variations in vector species and populations can often lead to differences in the functionality of protein products that can subsequently determine susceptibility and vector competence (Gubler et al., 1979; Tabachnick et al., 1985). Without access to genome sequences of all medically important species, studies in the past have relied on various genetic tools such as electrophoretic methods and quantitative trait loci mapping to determine the link between vector genetics and vector competence (Gomez-Machorro et al., 2004; Bennett et al., 2005). These pioneering studies and the information obtained from model organisms generated fundamental knowledge needed to discover genes and pathways that control the process and outcomes of arbovirus infections. The development of different molecular biological techniques in the late 20th century provided tools, for example RNA interference (RNAi), to examine individual factors as determinants of the outcome of arbovirus infection *in vivo*. Despite these advances and the availability of sequence data for several important species of mosquito, we still do not understand the basis of species specific susceptibility to virus infection and vector competence.

#### Genetic Approach to Study Vector Competence and Vector-Virus Interactions

The effort to determine the role of vector genetics on arbovirus susceptibility originally focused on the study of vector competence of various mosquito populations for arboviruses and the impact of selection during mosquito colonization on vector competence. By targeting the variable genetic loci encoding several metabolic enzymes, the impact on *Ae. aegypti* genetics during the process of colonization was first shown to be linked with their susceptibility to YFV (Lorenz et al., 1984). This observation was subsequently examined using comparative studies on the susceptibility of two subspecies of *Ae. aegypti*, *Ae. aegypti aegypti*, and *Ae. aegypti formosus*, to other flaviviruses, dengue virus, and yellow fever virus (Tabachnick et al., 1985; Vazeille-Falcoz et al., 1999). Using a quantitative genetics approach *Ae. aegypti formosus*, which is restricted to sylvan and rural areas of West Africa, was shown to have significantly lower susceptibility to human arboviruses due to genetic differences from its related competent vector species, *Ae. aegypti aegypti* (Bosio et al., 1998). Although this topic continues to be actively investigated in other studies involving vector competence of different vector populations for urban and sylvatic strains of DENV and YFV (Dickson et al., 2014), a substantial challenge has emerged as some *Ae. aegypti formosus* have become domesticated (Brown et al., 2011). For example, in an outbreak caused by DENV-3 in Cape Verde, an archipelago of 10 islands off the Atlantic coast of West Africa, *Ae. aegypti formosus* was reported to be a competent vector that is highly susceptible to YFV and CHIKV, which are both regarded as re-emerging arboviruses with high public health significance (Vazeille et al., 2013). Despite much effort, and availability of some mosquito genomes, the genetic basis of species and population-specific mosquito susceptibility/refractoriness to infection, genetic determinants of dissemination and of transmission are still poorly understood.

#### Innate Immunity of Arthropod Vectors and Arbovirus Infection

With evidence indicating that mosquito genetics can influence susceptibility and competence for arboviruses, the next critical step was to identify pathways and specific genes that act as determinants for the infection process. The early search for determinants of vector competence focused on factors acting as immune components in mosquitoes and largely benefitted from the pioneering studies conducted on *Drosophila melanogaster*, a model organism used for studies on innate immunity and pattern recognition receptors (Hoffmann, 2003). One of the well-characterized pathways discovered was the antiviral RNAi response that was originally discovered and characterized in fruit flies (Galiana-Arnoux et al., 2006). Both fruit flies and mosquitoes share this conserved mechanism which detects the presence of RNA viruses by detecting double-stranded RNA which triggers the RNAi response. The presence of double-stranded RNA indicates viral replication in infected cells (Westaway et al., 1997; Li et al., 2002). The development of an *in vivo* RNAi mediated knockdown system utilizing the Sindbis virus expression system, in *Ae. aegypti* mosquitoes, was initially used to knockdown a variety of endogenous mosquito genes and viral genes *in vivo*. This system effectively reduced DENV-2 and YFV transmission in *Ae. aegypti* (Olson et al., 1996; Higgs et al., 1998), reduced luciferase expression in transgenic *Ae. aegypti* (Johnson et al., 1999), and knockdown of a GATA factor which functions as a repressor gene in blood feeding *Ae. aegypti* mosquitoes (Attardo et al., 2003). Utilizing a similar alphavirus based o’nyong-nyong virus expression system, the importance of RNAi in limiting the replication of a medically important arbovirus in mosquitoes was demonstrated in *Anopheles gambiae* infected with o’nyong-nyong virus (Keene et al., 2004). Additional supportive evidence for the importance of RNAi in limiting arbovirus infections was obtained through additional experiments using DENV and Sindbis virus in *Ae. aegypti* (Campbell et al., 2008; Sanchez-Vargas et al., 2009; Khoo et al., 2010). Interestingly, the antiviral RNAi response in mosquitoes is also modulated by environmental conditions, especially extrinsic temperature. Cooler temperatures may increase the susceptibility of mosquitoes to arboviruses by impairing the RNAi response (Adelman et al., 2013).

As more data suggests that RNAi can mediate potent antiviral responses in mosquitoes, strategies utilized by arboviruses to escape the immune response have also been discovered. Through the expression of subgenomic RNA molecules, several arboviruses have developed an efficient strategy to ensure the ability to replicate by sequestering the antiviral RNAi machinery (Moon et al., 2015). In contrast to the significant advancements made in mosquitoes, the importance of RNAi-mediated antiviral responses is less well understood in ticks. Evidence from *in vitro* studies have shown that replication of Langat virus also triggers the RNAi response in *Ixodes scapularis* ticks, most likely through similar mechanisms as are found in mosquitoes (Schnettler et al., 2014). Despite observations that indicate RNAi plays an important role in arthropod antiviral immunity and the initial success in using RNAi to develop control strategies for arboviruses (Franz et al., 2014), the use of RNAi as a control measure remains limited. For example, a genetically engineered RNAi response targeting DENV failed to consistently maintain resistance in transgenic mosquitoes (Franz et al., 2009, 2014). The future of exploiting the RNAi response to produce pathogen-resistant arthropods remains unclear. However, the results thus far with RNAi are similar to other findings that mosquitoes rely on multiple mechanisms to defend against infection by arboviruses. For example, o’nyong-nyong virus replication is impeded by the heat shock protein 70B in *Anopheles gambiae* (Sim et al., 2007). The Toll, IMD, and Jak-STAT pathways have been shown to be activated in response to Sindbis virus and DENV in *Ae. aegypti* and WNV in *Culex pipiens* (Xi et al., 2008; Luplertlop et al., 2011; Zink et al., 2015). Interestingly, while arthropod vectors develop immune responses to limit viral infection, arboviruses have also developed what may be regarded as immune-escape strategies. This becomes evident by further characterization performed with CHIKV and Semliki Forest virus that showed infection of alphaviruses results in the suppression of signaling pathways (Fragkoudis et al., 2008; McFarlane et al., 2014).

#### Genomic and Transcriptomic Tools to Characterize the Vector-Virus Interactions

In addition to studies focused on immune responses, the broader picture of how arthropod vectors interact with arboviruses was further clarified once tools became available to study genomes and transcriptomes. Progress was particularly evident for studies on medically important mosquito species with available genomic sequences. Through the characterization of transcriptome responses, it became clear that the infection process of arboviruses involves complex responses related to detoxification, metabolism, immunity, DNA replication, protein translation, and apoptosis (Sanders et al., 2005; Girard et al., 2010; Tchankouo-Nguetcheu et al., 2010; Colpitts et al., 2011). Several genes identified in these transcriptome studies have been further characterized to determine the importance of arbovirus infections in arthropod vectors. Because of its importance in limiting virus infections in vertebrate hosts, apoptosis signaling pathways were evaluated in a number of studies; however, the role of apoptosis in arbovirus infection in arthropod vectors remains contradictory (Wang et al., 2008, 2012; O’Neill et al., 2015; Eng et al., 2016). This may be due to the fact that approaches used to knockdown individual caspases and inhibitors failed to consider the hierarchical organization and complexity of apoptotic pathways.

In addition to the concern that the experimental approach of manipulating individual genes may not produce conclusive and biologically relevant results, there is evidence indicating that different components of a physiological response such as digestion, nutritional status, and reproductive status may be involved in both the defense mechanisms of arthropod vectors and also play a critical role in the arboviral life cycle (Wikel et al., 2017). Such complexity is well exemplified by studies showing the ubiquitin proteasome pathway is involved in the arthropod immune response and release of infectious viruses. By monitoring and manipulating the expression of a mosquito ubiquitin protein, Ub3881 in various tissues, its antiviral function was demonstrated by labeling the DENV envelope protein for degradation as an immune response to limit viral infection (Troupin et al., 2016). In addition to its role in innate immunity, the ubiquitin proteasome pathway is also critical for the release of DENV particles from an infected midgut of *Ae. aegypti* (Choy et al., 2015). Although these studies have identified several cellular factors contributing to the susceptibility and refractoriness of specific arthropod vectors to arboviruses, it has become clear that vector competence is likely to be determined by multiple pathways in arthropods. While manipulation of individual genetic products remains the only feasible approach to characterizing vector-virus interactions, the broad view on how different pathways and their interactions can influence vector competence for arboviruses is still needed for improved mechanistic understanding of the infection, dissemination, and transmission processes of arboviruses in arthropod vectors.

### Viral Genetics and Arbovirus Infections in Arthropod Vectors

Whilst the manipulation of individual genes in arthropod vectors is helpful for identifying genes and pathways that are involved in the arboviral infection process, the other aspect of vector-virus interactions is the virus. Virus mutants with distinct phenotypes have been used to infect arthropods in order to identify the relationship between viral sequence and phenotype (McElroy et al., 2005; Anderson and Rico-Hesse, 2006; Kenney et al., 2012). This approach has led to substantial advancements in the field of arbovirology due to the low fidelity during the replication of the RNA genome among medically important arboviruses in the families of *Flaviviridae*, *Togaviridae*, and *Bunyaviridae* leading to rapid viral genetic changes (Xia et al., 2016). In nature, the evolution of arboviruses has repeatedly resulted in changes in epidemic potential and transmission patterns (Davis et al., 2005; Schuffenecker et al., 2006; Schuh et al., 2014). Several of these examples were found to be associated with increased infectivity or transmission efficiency in arthropods which becomes the mechanism for the emergence and re-emergence of arboviruses (Moudy et al., 2007; Dubrulle et al., 2009). Characterization of viral mutants, either isolated from nature or derived from laboratory experiments, can aid in the characterization of arboviral genotypes that can alter the phenotype of arboviruses in arthropod vectors.

#### Yellow Fever Virus as a Model to Study Viral Genetics and Its Impact on Vector-Flavivirus Interactions

Although the techniques of determining genetic sequences of arboviruses did not become available until the 1980s, observations indicating that mutations result in different phenotypes and consequences of infection were reported prior to the genomic era. The earliest example in the literature may be the effects caused by the serial passage of YFV in chicken embryos originally used to generate the attenuated 17D strain. Although the genetic composition was unknown, the attenuated 17D strain was shown to be deficient in disseminating from the midgut of infected mosquitoes (Whitman, 1939; Miller and Adkins, 1988; McElroy et al., 2008). This phenotype was later demonstrated to be caused by differences in the genetic composition between the attenuated and virulent strains (McElroy et al., 2005, 2006a,b). The distinct phenotypes between virulent and attenuated strains of YFV in mosquitoes provide a unique model to characterize vector-virus interactions. The identification of individual determinants in the viral genome which control the outcome of infection in mosquitoes has not yet been achieved. However, knowledge derived from these earlier studies has led to several discoveries indicating that domain III of the flavivirus envelope protein of YFV, and of other flaviviruses, is a critical region for the determination of infectivity in arthropod vectors (Erb et al., 2010; Huang et al., 2014).

#### Emergence of West Nile Virus Genotypes and Its Public Health Significance in North America

Since the introduction of WNV into the United States in 1999, the study of viral genetics and its influence on virus-vector interaction in nature has been extensively carried out by genotypic and phenotypic analyses (Mann et al., 2013). The first significant increase in its clinical incidence and public health significance was attributed to the emergence of the North America/WN 2002 genotype. This genotype efficiently displaced the New York 1999 genotype with two major surges in the numbers of human infections with neurological diseases in 2002–2003 and 2012. Its selective advantage was later determined to be the reduced extrinsic incubation period in *C. pipiens* and *C. tarsalis* but not the dosage required for the establishment of infection in mosquitoes (Ebel et al., 2004; Moudy et al., 2007; Vanlandingham et al., 2008). While phylogenetic analyses suggest the change of phenotype is linked to the single V159A substitution in the envelope protein (Davis et al., 2005), direct experimental evidence, using a reverse genetics approach to characterize the substitution, is still missing to test the hypothesis that the gain of selective advantage in mosquitoes is due to the single mutation. In the last 20 years, primarily because of the availability and use of molecular methods, especially those based on Polymerase Chain Reactions (PCR), it has become possible to detect viruses in mosquitoes that were not revealed using traditional methods because they killed neither cell cultures nor suckling mice. A comprehensive review of these arthropod-specific viruses that do not infect vertebrates and are maintained by transovarial transmission was recently published (Calisher and Higgs, 2018). A question that has been asked is whether or not these viruses may influence vector infection with other closely related viruses. Potential interference with WNV was evaluated by Goenaga et al. (2015) and Hall-Mendelin et al. (2016).

#### The Relationship Between Chikungunya Virus Mutation and Vector Competence

Another example that viral genetics contributes to the epidemic potential of an arbovirus was reported in the re-emergence of CHIKV in the Indian Ocean in 2005 (Huang et al., 2018). Historically, CHIKV is known to be transmitted by *Ae. aegypti* in its endemic regions, especially Africa and Southeast Asia. The change of its primary vector species to *Ae. albopictus* was reported in an outbreak on several islands in the Indian Ocean (Reiter et al., 2006). With its rapid dispersal in the late 20th century, the species was also subsequently shown to trigger local transmission of CHIKV in Italy and France (Angelini et al., 2007; Gould et al., 2010). Although mutations subsequently detected in other regions of its genome may contribute to this process, the adaptation of CHIKV to *Ae. albopictus* was primarily driven by the acquisition of the A226V mutation in its E1 protein (Tsetsarkin et al., 2007; Tsetsarkin and Weaver, 2011). Surprisingly, despite the fact that the selective advantage in transmission created by the E1-A226V mutation in the East-Central-South-African genotype is maintained in several epidemics recorded in different geographic regions (de Lamballerie et al., 2008), the introduction of CHIKV into the New World in 2014 was caused by the dispersal of its Asian genotype, which lacks the A226V mutation. As the introduction and autochthonous transmission of the East-Central-South-African genotype was detected in Brazil in 2015 (Nunes et al., 2015), it is unclear if the A226V mutation will be selected through the transmission by *Ae. albopictus* in the New World.

#### Genetic Reassortment of Bunyaviruses and Vector-Virus Interactions

In contrast to genetic drift created by accumulation of individual mutations in viruses containing single-stranded RNA genomes, genetic shift is attributed to the reassortment of segmented genomes (Briese et al., 2013). Segment reassortment can potentially cause a large number of genetic changes in an arbovirus. Among the medically important arboviruses, the process of genetic reassortment is unique to viruses in the family *Bunyaviridae* (Briese et al., 2013). Segment reassortment can occur if either the vertebrate host or the arthropod vector is simultaneously infected with two or more related viruses. In nature, reassortant bunyaviruses have been previously isolated from both vertebrate hosts and arthropod vectors (Klimas et al., 1981; Reese et al., 2008). This process has been reported to contribute to the emergence of new and more virulent viruses. A reassortment event in 1997 and 1998, between Bunyamwera virus and Batai virus (BATV) resulted in the emergence of Ngari virus (NRIV), a reassortant *orthobunyavirus*, which caused a large viral hemorrhagic fever outbreak in several East African countries, including Kenya and Somalia (Gerrard et al., 2004; Briese et al., 2006). NRIV contains the S and L segments of Bunyamwera virus and the M segment of BATV. Because the M segment encodes the structural genes of BATV, the virion of NRIV is likely to share similar structure and biochemical properties of BATV. NRIV was found to infect *Anopheles* species mosquitoes which are also the likely vector species for BATV (Huhtamo et al., 2013; Ochieng et al., 2013; Liu et al., 2014; Odhiambo et al., 2014). The finding demonstrates that the structure genes encoded in the M segment are likely to contain the determinants for vector range, infectivity, and efficiency of developing the disseminated form of infection in arthropods, as previously reported over 30 years ago (Beaty et al., 1981, 1982; Sundin et al., 1987).

The mechanisms controlling virus genetic reassortment are not completely understood. Characterization of the dynamics of reassortment has been investigated using mosquitoes fed two La Crosse virus strains in order to induce homotypic reassortment (Beaty et al., 1985). Interestingly, the incidence of dual infection and presumably the subsequent genetic reassortment significantly increases if two viruses are ingested simultaneously or separately with an interval less than 48 h (Beaty et al., 1985). Although the conclusion from the La Crosse virus dual infection studies are different from another superinfection model based on dual infections of the alphavirus, CHIKV, and the flavivirus, DENV (Nuckols et al., 2015), these studies demonstrate that superinfection of multiple arboviruses is likely to occur through the ingestion of viremic blood meals. Although the dynamics of superinfection has been previously studied, another important question that remains unaddressed is the packaging mechanisms of the three genomic segments to generate reassortant viruses. It is generally believed that the packaging of the three genomic segments is not a random process and requires complex interactions between viral RNA and nucleoproteins in order to generate a reassortant bunyavirus (Pringle et al., 1984; Hornak et al., 2016).

### Infection With Symbiotic Bacteria and Vector-Virus Interactions

The role of symbiotic bacteria in persistently infected mosquitoes has become increasingly studied and understood in vector-virus interactions (Wikel, 1996; Higgs, 2004). There have been several species of symbiotic bacteria that have been found to interfere with the infection of arboviruses in arthropod vectors. A particularly good example is the infection of *Wolbachia* in mosquitoes that can lead to a decrease in vector competence for arboviruses (van den Hurk et al., 2012). Persistent infection of *Wolbachia* has been reported in all arthropod species except for *Ae. aegypti*. Species of *Wolbachia* have been evaluated for their potential to suppress the vector population due to the discovery that artificially infected *Ae. aegypti* can lead to cytoplasmic incompatibility and embryonic lethality when mated with uninfected *Ae. aegypti* in nature (Hoffmann et al., 2011). *Wolbachia*-based vector control strategies have been shown to suppress the replication of WNV in its vector, *C. quinquefasciatus* (Glaser and Meola, 2010). Other studies have also demonstrated its interference in the replication and transmission of DENV, CHIKV, and Zika virus in *Ae. aegypti* (Walker et al., 2011; van den Hurk et al., 2012; Aliota et al., 2016); however, the bacteria did not show any significant reduction of vector competence of *Ae. aegypti* for YFV and *C. tarsalis* for WNV (van den Hurk et al., 2012; Dodson et al., 2014).

Several mechanisms have been proposed to explain the resistance to arboviruses induced by *Wolbachia* infection. The majority of available evidence suggests *Wolbachia* infection leads to the activation of immune responses and limits viral replication (Terradas and McGraw, 2017). However, mechanistic evidence regarding the pathways and effector genes is still needed (Bian et al., 2010; Pan et al., 2012; Rances et al., 2012, 2013). With its success in limiting arbovirus replication in *Ae. aegypti*, it is no surprise that this approach was further evaluated as a control strategy for *Ae. albopictus* (Moretti et al., 2018), a highly invasive vector species competent for various arboviruses. Although initial evidence suggested that *Ae. albopictus*, artificially infected by an *Wolbachia* strain derived from *Drosophila* under laboratory conditions, can interfere with infection of CHIKV and DENV (Blagrove et al., 2012, 2013), infection of *Wolbachia* among *Ae. albopictus* in nature did not lead to any significant impairment of infection and dissemination of CHIKV (Ahmad et al., 2017). As *Wolbachia* infection triggers a different set of physiological responses in *Ae. albopictus*, particularly immune responses, further evaluation is needed before a comprehensive understanding of what impact *Wolbachia* infection has on infection of arboviruses in *Ae. albopictus* (Molloy and Sinkins, 2015).

In contrast to the impact of the artificial introduction of *Wolbachia* into medically important mosquito species, another approach focuses on characterizing the interactions among arthropod vectors, arboviruses, and symbiotic bacteria. This type of interaction has been shown to be particularly important to the susceptibility of mosquitoes to arboviruses. For example, the colonization of microbiota in the midgut of *Ae. aegypti* triggers basal immune responses that suppress the infection of DENV (Ramirez et al., 2012). Although the majority of the bacterial population in a mosquito midgut and their interactions remains to be investigated, it has already become clear that specific species may have an inhibitory effect, not only to arboviruses, but also to other pathogenic microorganisms (Ramirez et al., 2014).

## Vector-Host Interactions and Their Impact on Transmission and Disease Pathogenesis of Arboviruses

Arbovirus infections are established through the feeding of arthropod vectors on vertebrate hosts, a much more complex process than the simple inoculation of arboviruses using, for example, needle inoculation. This natural feeding process creates complicated interactions between arthropod vectors and vertebrate hosts as arboviruses are injected through the saliva, which quickly elicits responses to the feeding process from the vertebrate hosts. Depending on the duration of feeding and quantities of arboviruses delivered, non-viremic transmission of arboviruses among co-feeding arthropod vectors can also be created in at least two types of medically important arthropods, namely ticks and mosquitoes. Therefore, such interactions have become important factors in determining the transmission patterns and disease pathogenesis of arboviruses. This is discussed in more detail below.

### Roles of Arthropod Saliva in Disease Pathogenesis

A recent review by Higgs et al. (2017), describes mosquito modulation of arbovirus-host interaction, whilst Higgs and Vanlandingham (2016) compare and contrast ticks and mosquitoes with an emphasis on tick-borne transmission. Although many of the proteins present in mosquito saliva have unknown functions, there are others that are known to alter normal physiological functions of the host which facilitates the acquisition of blood meals. The experimental transmission of arboviruses in animal models has shown that saliva leads to more severe disease than virus inoculation without saliva (Schneider and Higgs, 2008). Functional characterization has demonstrated multiple roles of saliva in vasodilation, pain suppression, anti-inflammation, anti-coagulation, and anti-hemostasis. Pathogen transmission with saliva has also been found to alter host immune responses. These functions often are associated with the increase of viral replication and dissemination in vertebrate hosts and the augmentation of pathological outcomes mainly by compromising the host immune mechanisms.

In several *in vitro* experimental systems, the growth of arboviruses can be promoted by the addition of salivary components or their extracts, which suppress the production of antiviral cytokines (Fuchsberger et al., 1995; Hajnicka et al., 1998; Limesand et al., 2003). Although the cell lines chosen in these studies may not necessarily reflect the tissue tropisms of arboviruses, it has become evident that the immunomodulation caused by arthropod saliva is critical for disease pathogenesis of different arboviruses. In spite of the differences in the feeding processes between ticks and mosquitoes, the presence of salivary gland components from both species has been shown to cause enhancement in disease severity of several arboviruses belonging to different virus families in different animal models (Dessens and Nuttall, 1998; Edwards et al., 1998; Limesand et al., 2000; Schneider et al., 2006; Hermance and Thangamani, 2015). While the species of vertebrate hosts are different in various studies, the immunomodulation properties of saliva from mosquitoes generally promote the T_H_2 immune response and prevents the clearance of arboviruses and other intracellular pathogens transmitted by arthropods, which is mainly driven by the T_H_1 immune response (Kovar et al., 2002; Schneider et al., 2004; Skallova et al., 2008; Thangamani et al., 2010; Cox et al., 2012). However, the induction of the T_H_2 response is less apparent in the feeding of *I. scapularis* ticks infected by POWV, indicating the fundamental difference in the saliva-mediated immunomodulation between mosquitoes and ticks (Hermance and Thangamani, 2014). Using WNV as a model, the immunomodulation by mosquito saliva can also be shown by the change in the recruitment process of T lymphocytes to the site of infection and ultimately modulate the antigen presentation cell signaling critical for the dissemination of arboviruses (Schneider et al., 2010). The increased recruitment of neutrophils and monocytes and antigen presentation cell migration to draining lymph nodes can also be induced when mosquito salivary gland extracts and DENV were delivered through needle inoculation (Schmid et al., 2016). In addition to the change in the local tissues and lymph nodes, the effect induced by the salivary gland components was also demonstrated to be systemic. When challenge with Rift Valley fever virus was performed in the presence of salivary gland extract, significant increase in viral load of blood, liver, and brain was observed (Le Coupanec et al., 2013). Whilst the feeding process of mosquitoes and ticks are remarkably different, there has been a significant level of similarity reported in the immunomodulation caused by tick and mosquito saliva. The stimulation in the cell recruitment process by tick saliva has also been found to be important for disease pathogenesis of tick-borne arboviruses. During the prolonged feeding period of ticks, phagocytes and neutrophils were reported to be the primary cell types recruited to the feeding sites in the very early stage of feeding from POWV-infected ticks. Interestingly, the same study also indicated that macrophages and fibroblasts are likely to be the primary cell types infected by POWV during the early stage, although the establishment of infection coincided with the activation of pro-inflammatory cytokines (Hermance et al., 2016). Interestingly, in spite of the similarity in the stimulation of cell recruitment process by mosquito and tick saliva, changes in the cytokine expression profile induced by tick saliva did not reveal the preferential change toward the T_H_2 response. The production of pro-inflammatory cytokines was triggered in mice exposed to ticks infected by POWV (Hermance and Thangamani, 2014). The observation may be a reflection of the fundamental difference in the cellular and molecular interactions triggered by saliva between mosquito and tick feeding. Such knowledge may be critical for our understanding of the establishment of infection and disease pathogenesis of mosquito-borne and tick-borne arboviruses in vertebrate hosts.

With the increased understanding of the dynamics of immunomodulation caused by arthropod saliva, several studies have been designed to determine the specific functions of individual salivary components. Targeting the innate and adaptive immune responses, there have been several known mechanisms that cause the enhancement of arbovirus infection in the presence of arthropod saliva. Type-I interferon (IFN) and its downstream signaling pathways, one of the most important mechanisms in innate antiviral immunity, can be impaired at different levels by salivary proteins. Several molecules in the saliva of *Ae. aegypti* have been shown to suppress the type-I IFN signaling pathway *in vitro* by suppressing the mRNA expression of type-I IFN, IFN-responsive element, and effector genes (Surasombatpattana et al., 2014). Similar suppression of type-I IFN was also observed with the treatment of tick salivary gland extract with detailed mechanisms to be determined (Hajnicka et al., 1998). In contrast to the shutdown of innate immune signaling that mediates broad-spectrum antimicrobial immunity, adaptive immune responses can also be compromised by salivary protein components of arthropods. For example, the significant increase of DENV viremic titers is associated with the reduced level of cytokines related to adaptive immune responses caused by aegyptin, an *Ae. aegypti* salivary protein (McCracken et al., 2014). In addition to the impairment of immune responses, other novel pathways have also been suggested. Through the secretion of a serine protease molecule, mosquito saliva has been shown to promote the infection of DENV by increasing binding affinity to cellular receptors such as proteoglycan molecules and the induction of cell migration, which can potentially facilitate the dissemination of virus (Conway et al., 2014).

With our increased understanding of the importance of salivary proteins in the enhancement of arbovirus infection, it has become apparent that the salivary components of arthropods are critical for the pathogenesis of arboviruses. Such knowledge may later become helpful in the development of animal models for disease research and strategies for intervention and prevention of severe diseases. However, our readers must keep in mind that the results must be interpreted with caution as the modulation of immune responses and enhancement of infection are often observed in laboratory animals that are not natural hosts for most arboviruses.

### Vector-Host Interactions and Arbovirus Transmission

In addition to the enhancement of arbovirus infection, interactions between arthropod vectors and vertebrate hosts can also be deciding factors for transmission (Higgs and Vanlandingham, 2016; Higgs et al., 2017). As the vectors inject infectious viruses into vertebrate hosts, the process transiently creates a unique environment that contains high concentrations of arboviruses and can promote an alternative but important mode of transmission called non-viremic transmission. Such a process occurs when feeding of infected and susceptible arthropod vectors takes place simultaneously in close proximity to each other on a vertebrate host. The term non-viremic transmission was initially used to describe this mode of transmission because of the lack of detectable viremia in a vertebrate host, and can also be referred to as non-replicative transmission. The process promotes the transmission of some arboviruses as it may not necessarily require a viremic vertebrate host. Through the creation of a local environment that contains a high viral load, this mode of transmission can happen in vertebrate hosts that are resistant or not permissive for viral replication to develop a viremia of a magnitude that exceeds the threshold titer that is generally assumed necessary to facilitate transmission.

Non-viremic transmission was described in Thogoto virus, an orthomyxovirus transmitted by the African brown ear tick *Rhipicephalus appendiculatus* in a guinea pig model (Jones et al., 1987). Whilst guinea pigs normally do not develop high viremic titers to sustain Thogoto virus transmission, transmission was found to occur through co-feeding of infected adults with uninfected adults or nymphs on the same guinea pig without detectable viremic titers. As ticks often feed for a prolonged period on vertebrate hosts, it is no surprise that this type of transmission can significantly increase during a longer co-feeding process which enables longer exposure to infectious viruses (Jones and Nuttall, 1989). *R. appendiculatus* has been shown to be more efficient than *Amblyomma variegatum* as donor ticks in mediating non-viremic transmission. This finding indicates that non-viremic transmission may take place during co-feeding of certain tick species at higher incidence (Jones et al., 1990). The characterization of non-viremic transmission was quickly expanded to the characterization of transmission and maintenance mechanisms for TBEV, a zoonotic flavivirus that is maintained in wild animals, especially rodent species (Labuda et al., 1993a,b). Interestingly, in addition to non-viremic transmission taking place in vertebrate hosts resistant to TBEV infection, non-viremic transmission can also have significant influence on the dynamics of TBEV transmission and evolution in susceptible vertebrate hosts. Non-viremic transmission through co-feeding *I. ricinus* has a significantly higher incidence in vertebrate hosts that are susceptible to TBEV (Labuda et al., 1993c). More importantly, this mode of transmission can also occur in vertebrate hosts which have immunity against TBEV (Labuda et al., 1997). The finding suggests non-viremic transmission can take place for a longer period of time and be independent of viremia. It has several important impacts on the evolution and spread of tick-borne arboviruses. This process can result in the selection of avirulent strains with delayed onset of viremic phase and low mortality in vertebrate hosts. Non-viremic transmission is also advantageous for viral transmission because it allows ticks to complete the engorgement process. This hypothesis is also supported by the finding that genetic elements of the European subtype of TBEV promote non-viremic transmission by showing a higher incidence of non-viremic transmission in *I. ricinus* than the Siberian subtype (Khasnatinov et al., 2016). However, a significant gap of knowledge is the undetermined virulence of both subtypes in vertebrate hosts as natural reservoirs. An overview of non-viremic and salivary-activated transmission of tick-borne viruses is provided by Higgs and Vanlandingham (2016).

Whilst non-replicative or non-viremic transmission was mainly observed through the prolonged co-feeding of ticks, it was later demonstrated that non-viremic transmission can also take place in mosquito-borne arboviruses (Huang et al., 2017). Secretion of WNV at sufficiently high concentration by infected donor mosquitoes was shown to result in non-viremic transmission to uninfected recipient mosquitoes (Higgs et al., 2005). This process relies on a transient non-propagative environment with high viral load at the sites where co-feeding of multiple arthropods takes place (Reisen et al., 2007). Because the increased temporal and spatial separation in the feeding of donor and recipient arthropods is likely to reduce the availability of infectious viruses, the efficiency of non-viremic transmission is likely to be higher when arthropod vectors are co-feeding simultaneously in close proximity. However, transmission of WNV can also be achieved with temporal and spatial segregation (McGee et al., 2007). The finding further confirms the importance of this mode of transmission for the transmission of mosquito-borne viruses as it allows the transmission to occur in the sequential feeding of donor and recipient mosquitoes.

## Conclusion

Although our understanding of the interactions among arboviruses, arthropod vectors, and vertebrate hosts continues to improve, it is apparent that arthropod vectors do not merely serve as “mobile syringes” to deliver arboviruses to susceptible vertebrate hosts. Arthropods vectors, which actively mount immune and anti-viral responses to limit arbovirus infections, can provide a unique intracellular environment to select and promote the transmission of specific viral populations which can ultimately change the transmission patterns of arboviruses. Genetic engineering to enhance vector immune or anti-viral responses, or manipulation of the vector microbiome to reduce vector competence or suppress vector populations are all being considered as options to reduce the incidence of vector-borne viruses. Genetic approaches to modulate viral determinants of infection, dissemination, and transmission may help in the rationale design of safe and efficacious vaccine candidates that cannot be transmitted by arthropods. When feeding on vertebrate hosts, vector-host interactions have been proven to play a critical role in disease pathogenesis and further create alternative routes of transmission. Understanding these interactions have been shown to provide unique strategies for the development of animal models for disease pathogenesis. We can certainly expect that as our understanding continues to grow, new technologies will be developed. Combined with other findings in the field, a better understanding of these unique interactions will likely contribute to the formulation of more novel control strategies in the future. Applications of the relatively recently developed CRISPR technology, for example, not only offers a new method for genetic manipulation, but furthermore may be used as a gene drive to establish desirable traits such as resistance to infection in vector populations (National Academies of Sciences, 2016).

## Author Contributions

Y-JH, SH, and DV drafted and edited the article together.

## Conflict of Interest Statement

The authors declare that the research was conducted in the absence of any commercial or financial relationships that could be construed as a potential conflict of interest.
